# SURGICAL TREATMENT OF SEVERE OBESITY IN TEENS: LATE RESULTS

**DOI:** 10.1590/S0102-6720201500S10004

**Published:** 2015-12

**Authors:** Álvaro Antônio Bandeira FERRAZ, Luciana Teixeira de SIQUEIRA, Clarissa Guedes NORONHA, Danilo Belem Rodrigues de HOLANDA, José Guido Corrêa de ARAÚJO-JÚNIOR, Mariana Gomes MUNIZ

**Affiliations:** General Surgery Service at Hospital das Clínicas, Federal University of Pernambuco, Recife, PE, Brazil

**Keywords:** Obesity, Adolescents, Gastric bypass, Bariatric surgery, Complications

## Abstract

**Background:**

: In children is estimated that the prevalence of overweight and obesity has increased up to five times in developed countries and up to four in developing countries. In Brazil, the proportion of children and adolescents who are overweight also increased from approximately 4.1% to 13.9%.

***Aim*:**

To evaluate the surgical results of severe obesity in adolescents.

**Methods:**

: Retrospective descriptive study of 2737 patients with severe obesity that underwent Roux-en-Y gastric bypass selecting from the total 44 patients with mean age of 18.1 years, 14 males and 30 females, most (37) operated by laparotomy. There was follow-up of 20 patients (45.45%). All were followed preoperatively by a multidisciplinary team and had indication confirmed for surgical unanimous approval of all team members.

**Results:**

: Among the 20 adolescent, 14 were female. From five teenagers using anti-hypertension or hypoglycemic drugs before surgery, four (80%) had drug discontinuation and one (20%) reduced the dose in 50% postoperatively. The average weight loss was 45.4 kg after a mean follow up of 60 months. There were no deaths or severe postoperative complications. Among those who underwent postoperative follow-up with a multidisciplinary team, 18 were with BMI<30.

**Conclusions:**

: Adolescents undergoing Roux-en-Y gastric bypass has good response in relation to weight loss and improvement of comorbidities. There was a low rate of complications and no deaths. All patients were satisfied with their personal results.

## Abstract (pt)

**Racional:**

Em crianças estima-se que a prevalência de sobrepeso e obesidade aumentou até cinco vezes nos países desenvolvidos e até quatro naqueles em desenvolvimento. No Brasil, a proporção de crianças e adolescentes com excesso de peso também cresceu de aproximadamente 4,1% para 13,9%.

**Objetivo:**

: Avaliar os resultados cirúrgicos da obesidade severa em pacientes adolescentes.

**Métodos:**

: Estudo retrospectivo e descritivo de 2737 pacientes portadores de obesidade severa e submetidos à derivação gástrica em Y-de-Roux selecionando deles 44 com idade média de 18,1 anos. Houve seguimento ambulatorial de 20 pacientes (45,45%). Todos foram acompanhados pré-operatoriamente por equipe multidisciplinar e tiveram sua indicação cirúrgica confirmada após a aprovação unânime de todos os membros da equipe.

**Resultados:**

: Entre os 20 pacientes adolescentes 14 eram mulheres. Cinco do adolescentes em uso de anti-hipertensivos ou hipoglicemiantes antes da operação, quatro (80%) tiveram a suspensão da medicação e um (20%) reduziu a dose em 50% no pós-operatório. A média de perda ponderal foi de 45,4 kg após seguimento médio de 60 meses. Não houve mortalidade no grupo estudado nem complicações operatórias graves. Entre os que realizaram o acompanhamento pós-operatório com equipe multidisciplinar, 18 ficaram com IMC<30.

**Conclusões:**

: Adolescentes submetidos à derivação gástrica em Y-de-Roux tiveram boa resposta em relação à perda de peso e melhora das comorbidades. Apresentaram baixa taxa de complicações e nenhum óbito. Todos ficaram satisfeitos com os resultados.

## INTRODUCTION

Obesity is a problem that has worried the public health entities over the last years. Thus, there is a world interest in fighting weight excess among the population due to the comorbidities such as systemic arterial hypertension, type II diabetes mellitus, hypertrigliceridemia, sleep apnea, coronary disease and stroke^8^^,^ 14 ^,^ 19. 

Despite the endeavours undertaken by the scientific community and other members of the society, figures associated with obesity have been increasing along the last decades, especially in developing countries^5^^,^ 11 and in young populations^1^^,^ 3 ^,^ 23. There are approximately 300 million obese people in the world^8^.

In Brazil, more than 40% of the adults are overweighted. Today, obesity is the third nutritional disease of the country, losing only to anemia and malnutrition^21 ^. Between 1980 and 2000, it is estimated that the prevalence of obesity in children increased up to five times in developed and up to four in developing countries^17^^,^ 27. In Brazil, the proportion of overweighted children and adolescents grew from approximately 4.1% (1974/1975) to 13.9% (1996/1997)^27^.

In studies with adult people, it was seen that abdominal obesity is a factor associated with cardiovascular events and mortality^2^^,^ 13. In adolescents, the accumulation of abdominal fat has been pointed as a risk factor in the occurrences of cardiovascular and metabolic diseases^10^^,^ 12 ^,^ 25. In addition to that, increased abdominal adiposity is associated with the elevation of arterial pressure^20^, greater concentration of triglycerides and hiperinsulinemia^22^. 

Adolescence is a life period which deserves special attention, since the transition between childhood and adulthood may result, or not, in future problems for the individual's development. In this phase, many changes occur; both of physical and psychological nature, which make possible the appearance of irreverent behavior as well as questioning of models and children's standards, necessary for the development itself. Due to the psychological profile of this group of patients and, despite the endeavors of multidisciplinary teams (endocrinologists, nutritionists, psychologists and psychiatrists), many are not able to lose weight and maintain weight loss, so that, studies prove that 50-77% of these children remain obese in the adult life. In case there is some obese relative, the risk increases to 80%^7^. These data foster the performance of surgical procedure, since it makes possible the treatment of most adolescents who would not get better in terms of weight rates and would remain obese during the adult life and would also present morbidities. 

Brazilian Federal Council of Medicine - CFM's resolution n^o.^ 1.942/2010 alters resolution CFM's n^o.^ 1.766, of 13 May 2005, establishing safe norms for the surgical treatment of morbid obesity, defining indications, procedures and team. Thus, the surgery is indicated for patients over 18 and it mentions a minimum age of 16, with a warning regarding ponderation of risks as well as benefits. In elderly (over 65) and young patients between 16 and 18 years, the surgery can be performed whenever there are indication and consensus among family, multidisciplinary team - as long as special precautions are taken -, and a careful analysis regarding risk/benefit.

Thus, in these groups of people, there are benefits well clarified in the adoption of the treatment of Roux-en-Y gastric bypass (RYGB) in case of failure of the clinical treatment. It is the best way for efficient and long-lasting weight loss^4^^,^ 9. The indication should be very discerning, so that the benefit can be advantageous in relation to the possible disorders caused by morbid obesity. Such disorders include depression, difficulty in interpersonal relationship, affective and intellectual development, as well as cardiovascular and constitutional diseases^16^^,^ 24 ^,^ 28. 

Thus, the present paper intents to evaluate the surgical results of severe obesity in adolescents.

## METHODS

In the period from 2002 to 2011, 2.737 patients with severe obesity were submitted to Roux-en-Y (RYGB) gastric bypass by laparotomy or laparoscopy in the Service of General Surgery of Hospital das Clínicas, Federal University of Pernambuco, and in two private clinics in Recife, PE, Brazil. A retrospective descriptive study was carried out in a population of adolescents submitted to the procedure. According to the World Health Organization, adolescence comprehends the period from 11 to 19, involving body and physical changes arising from physical maturation.

After observation of inclusion and exclusion criteria, data were collected from medical charts covering the pre and postoperative periods, with the longest possible follow-up. Thus, 44 patients were evaluated (3.2%) with age ranging from 13 to 20, mean 18.1±1.49, of both genres, where 14 (3.2%) were male and 30 (68.9%) were female. The average body mass index (BMI) was 43.3±3.28, compared to 47.8±3.23 in the group over 20. The incidence of systemic arterial hypertension was 20.45% (9/44) and type 2 diabetes mellitus 13.63% (6/44). 

Thirty-seven were operated by laparotomy and seven by videolaparoscopy. Although 27 adolescents presented a postoperative follow-up greater than one year (average 28 months), only 20 were selected, based on the follow-up and history and recordings in the medical records, according to the following criteria: surgical technique, comorbidity and previous and present BMI, excluding the patients who lost clinical and outpatient or who refused to participate in the interview. 

The current data were obtained by means of interview in the office or by phone contact, as well as by charts research. A questionnaire was elaborated concerning the following variables: antropometric data (weight and BMI) in the pre and postoperative period; current use of medication and surgical complications defined as precocious (events related to the surgical act up to 30 days of postoperative period - superficial infection, deep or of organs and regions such as peritonitis or cavitary abscess, seroma of surgical wound, dehiescense of anastomosis and bleeding) or late (hernia of abdominal wall, malnutrition, ulcer of anastomotic mouth, dehiscence, anemia, colelithiasis, weight regain, anastomosis stenosis, dumping and gastrogastric or gastrojejunal fistula). 

Another criterium evaluated was the satisfaction with the surgical procedure. As well as the surgical team, endocrinologist, psychologist and nutritionist also participated in the study.

The data were typed into a Microsoft Office Excel 2010 database utilizing the Statistical Package for Social Sciences version 15, adopting the significance level of 5% for all the statistical tests (t-Student paired test, Pearson Chi-square or Fisher's exact) depending on the conditions of the sample.

## RESULTS

Out of the 44 adolescents who had been operated, there was an outpatient follow-up of 20 (45.45%), where 14 were women and six men. The average age was 18.25 (16-20). The BMI ranged from 37.61 to 52.85 (average 42.97±3.23).Thirteen patients (65%) presented with a BMI between 40-50; weight loss was seen in four of them (20%).

In the group of 20, the incidence of systemic arterial hypertension in the preoperative period in percentage of regular use of anti-hypertensive was 25% (n=5), and of type 2 diabetes mellitus 5% (n=1). Dislipidemia was seen in (25%), without drug treatment. 

According to Table 1, four out of the five patients using anti-hypertensive or hypoglycemic agents before the surgery had suspension of medication; and one had its dose reduced by 50% after surgery. The average weight loss was 45.4 kg after a follow-up ranging from one month to five years. Nine (45%) entered the normal range of BMI (<25) and another nine continued overweighted (BMI 25-30). Two were classified as grade 1 (BMI 30-35). Among the postoperative complications, seroma and ulcer of anastomotic mouth were the most prevalent, four (20%) in each complication. Iron deficiency was diagnosed in two (10%), with remission after one month anti-anemia treatment. Dumping, stenosis of anastomotic mouth and internal hernia were identified in one patient (5%) each, which were solved either by nutritional orientations, endoscopic procedures (four sessions of pneumatic dilations on average) and laparoscopy for closure of the Petersen space, respectively. There was no mortality in the group. 

When asked about their satisfaction with the surgical procedure, all patients answered positively regarding the surgical results. 

**TABLE 1:** - Relevant clinic characteristics of the patients (n=20)

**N°**	**Gender**	**Age at operation years**	**Pre BMI kg/m(2)**	**Weight loss Kg**	**Post BMI kg/m(2)**	**Medication usage before (SAH/DM)**	**Medication usage after (SAH/DM)**	**Complications**
1	F	20	42.24	50	23.87	yes	stopped	Iron defeciency
2	M	18	42.29	49	26.47	yes	reduced dose	none
3	F	16	42.68	38	28.19	no	no	none
4	F	18	39.13	38	23.3	no	no	seroma
5	F	18	42.15	51,8	22.65	no	no	dumping
6	F	18	41.8	55	23.83	no	no	none
7	M	18	42.66	62	24.54	yes	stopped	seroma, gastric ulcer
8	F	20	41.52	51	23.87	no	no	gastric ulcer
9	F	18	38.58	36	24.69	no	no	stenosis of anastomosis
10	M	19	42.41	42	28.04	yes	stopped	seroma
11	F	17	41.40	40	26.67	no	no	none
12	F	19	43.66	41	28.22	no	no	ulcer of anastomotic mouth, seroma
13	F	18	41.14	34	28.65	no	no	iron deficiency
14	M	17	52.85	72	31.35	yes	stopped	none
15	M	19	46.29	70	24.69	no	no	none
16	M	18	39.79	34	28.4	no	no	internal hernia
17	F	19	39.55	20	32.29	no	no	none
18	F	17	39.38	47	24,21	no	no	none
19	F	18	37.61	35	25.64	no	no	ulcer of anastomotic mouth
20	F	20	42.24	35	29.38	no	no	none

BMI=body mass index; F=female; M=male; DM=diabetes mellitus; SAH=systemic arterial hypertension

## DISCUSSION

Obesity is a risk factor for cardiovascular diseases, arthropathies and obstructive sleep apnea, which reduce life quality and expectancy by around 22%^6^, predominantly in younger groups. At this age range, there is an increase in cardiovascular risk, even in the asymptomatic. It was shown that the obese adolescent has abnormalities in the structure and cardiac functioning, such as left ventricular hypertrophy (60%), left ventricular dilation (50%) and increased pericardial adipose tissue^24^. 

On the other hand, obesity brings with it other limitations to the adolescent's life from the psychosocial perspective. Approximately 30% of obese meet the criteria for depressive symptoms clinically relevant based on self-evaluation and 45% on their mother's evaluation. However, only 21% are involved in some psychologic treatment^16^. 

Since there are problems related to obesity in schools at this age range, another study using this same group of patients will be able to evaluate the psychological aspects related to social acceptance before and after bariatric surgery^18^. Is it the case that the condition of non-obesity in the postoperative will increase the acceptance and social inclusion? 

Therefore, since the serious and immediate consequences of obesity in young people are more and more outlined, there is an increase in the acceptance of an earlier surgical treatment as the best way to reverse damages caused by such these diseases and the most effective treatment and of long lasting results for weight loss leading to improvement or remission of most comorbidities^6^. On the other hand, caution is necessary in the indication of the surgical treatment in this group, for this same psychological aspect might have an influence in frustration after surgery, due to the fact that a new pattern of behavior (physical activities) and eating habits is to be accepted with multidisciplinary follow-up as a routine, which may explain the lack of adhesion to the surgical treatment, with discontinuation of postoperative follow-up, as observed in this study (24 of 44). It is possible to notice, therefore, a group of patients without follow-up, who develop other health problems, such as anemia, malnutrition and weight regain. 

Another questioning is about cost/benefit of any surgical technique. For example, it is unknown how bariatric surgery performed before puberty conclusion and epiphysial fusion will affect neuroendocrinal, skeletal and psychosocial maturation^15^. In relation to RYGB, this questioning should also take into consideration the eating and behavioural changes of young people from all over the world, which have led to the evident growth in children and youth obesity cases. 

Thus, the adolescent patients, in order to be submitted to bariatric surgery, should be judged based on more rigid selection criteria. Initially, there have to be well organized attempts of weight loss for more than six months; patients should be psychologically mature and be able to adhere to the nutritional orientations in the postoperative period. They should also demonstrate ability to decide and knowledge of regarding the need of psychological and medical follow-up after the operation; agree in avoiding pregnancy for one year and have family support and involvement^15^. 

Based on these information, the study in question did a retrospective evaluation of this group of patients regarding the safety and efficacy of the bariatric method which was proposed. As well as comparing the concrete indexes such as ponderal loss, clinical improvement of associated morbidities and surgical complications, it was also asked an important factor which is the satisfaction with the global result of the intervention.

Among the most utilized and well studied surgical procedures for bariatric surgery are the adjustable gastric band and RYGB. The band has as its main hindrance being counter indicated by the US Food and Drug Administration in patients under 18. All patients involved in this study were submitted to RYGB, since it showed to be the most efficient method of weight loss in retrospective studies^15^^,^ 18. The percentage of weight regain in five years with the band is 40% and in RYGB is 10-20%^9.^ The efficacy of the method regarding weight regain was attested in the studied group (average of 45.4 kg after an average follow-up of 60 months). Nine patients (45%) entered the normal BMI range (<25) and other nine remained with overweight (BMI 25-30). Only two were classified as grade I (BMI 30-35). Although not having statistical relevance, from among the patients who were hypertensive before the surgical procedure and who achieved the normal BMI range, all of them returned to normal pressure levels, there being clinical relevance in the present study. 

Despite the efficacy regarding the weight loss maintenance, RYGB is not exempt of late sequelae. The most common are: incisional hernia when the technique is laparotomy (5-10%) and the complications related to the ring (stenosis or erosion). In the studied group, seroma and ulcer of anastomotic mouth were the most prevalent, being present in four (20%) each. Incisional hernia was not observed in the sample. The most worrying late sequelae are the nutritional ones, such as anemia, hipoalbuminemia, mineral and vitamin deficiencies, although anemia was diagnosed in only two patients, solved with anti-anemic treatment for one month. For these reasons, routine vitamin-mineral supplements are utilized; and it is necessary to monitor the nutritional state for life, by means of periodic exams and multiprofessional follow-up^6^. 

Thus, there are benefits, considering the low complication rate, especially when precautions are taken. Malnutrition continues to be the main postoperative alteration, followed by weight regain. Malnutrition has occurred in low percentage (<5%) in adults; and, since studies show that RYGB results adolescents are similar to those in adults, it is expected that the low incidence of complications also occur in the first group. 

However, despite the good results observed in this study, a follow-up has to be done with larger sample and time so that it come to be statistically relevant, since the loss of outpatient follow-up was considerable. This fact may be understood by the psychological immaturity at this age range.

There is good evidence that patients submitted to this procedure and who reach the normal range of BMI have better response regarding comorbidities. The loss of postoperative follow-up hinders the evaluation with better statistical significance. It is of fundamental importance to have a multidisciplinary postoperative follow-up in order to assure the maintenance of surgical results.

## CONCLUSION

Adolescents submitted to Roux-em-Y gastric bypass had good response in terms of weight loss and recovery from comorbidities. They showed low complication rate and no mortality. They all approved the results of the procedure.
